# 5-Aza-2’-deoxycytidine in the medial prefrontal cortex regulates alcohol-related behavior and Ntf3-TrkC expression in rats

**DOI:** 10.1371/journal.pone.0179469

**Published:** 2017-06-14

**Authors:** Xiaomeng Qiao, Fangyuan Yin, Yuanyuan Ji, Yunxiao Li, Peng Yan, Jianghua Lai

**Affiliations:** 1 College of Forensic Science, School of Medicine, Xi’an Jiaotong University, Xi’an, Shaanxi, China; 2 Key Laboratory of Environment and Genes Related to Diseases, Ministry of Education, Xi’an, Shaanxi, China; Oregon Health and Science University, UNITED STATES

## Abstract

Recent studies have indicated that DNA methylation plays an important role in the development of alcohol abuse. 5-Aza-2’-deoxycytidine (5-Aza-dc), an inhibitor of DNA methyltransferases, was FDA approved for myelodysplastic syndrome treatment. However, it is unclear whether 5-Aza-dc is involved in alcohol abuse. In this study, using a chronic alcohol exposure model in rats, 5-Aza-dc was injected into the medial prefrontal cortex (mPFC). Alcohol-drinking behavior and the anxiety related behavior were evaluated by two-bottle choice and open field test. We found that 5-Aza-dc injection into the mPFC significantly decreased alcohol consumption and alcohol preference in alcohol-exposure rats, corresponding to the reduced blood alcohol levels. Although 5-Aza-dc potentiated the anxiety-like behavior of alcohol-exposure rats, it had no effect on the locomotor activity. Moreover, both of the mRNA and protein levels of DNA Methyltransferase 3A (DNMT3A) and DNMT3B in the mPFC were upregulated after 35 days of alcohol exposure and this upregulation could be reversed by 5-Aza-dc treatment. Additionally, 5-Aza-dc reversed the alcohol-induced downregulation of neurotrophin-3 (Ntf3), correspondingly the expression of its receptor-TrkC was reduced. These findings identified a functional role of 5-Aza-dc in alcohol-related behavioral phenotypes and one of the potential target genes, Ntf3. We also provide novel evidence for DNA methyltransferases as potential therapeutic targets in alcohol abuse.

## Introduction

Alcohol abuse is a complex disorder that results in tolerance, withdrawal, relapse and cognitive deficits such as learning and memory impairment[1, 2], which are based on long-lasting gene expression and neuronal synaptic plasticity [3, 4]. DNA methylation can maintain long-term stability of related phenotypes by regulating the permanent silencing of specific genes [5, 6]. While several studies support a role of DNA methylation in regulation of alcohol-related behavior [7–9], but much less is known about the exact mechanism.

The prefrontal cortex (PFC) is associated with the regulation of cognitive, emotional, and motivational processes [10–12]. We focused on the medial prefrontal cortex (mPFC) because of its prominent role in drug-induced neuroadaptation associated with drug seeking and alcohol abuse. Recent results have indicated a significant association between mPFC function and drug addiction. For example, recordings of single mPFC neuronal activity during cocaine and heroin self-administration reveal a strong behavioral association by which a substantial number of mPFC neurons are modulated (excited or inhibited) at different phases during drug seeking behaviors [13]. Chronic alcohol consumption is associated with executive dysfunction and with changes in grey and white matter volume in the mPFC [14]. In addition, escalated alcohol intake was associated with increased DNA methylation and decreased expression of genes encoding synaptic proteins involved in neurotransmitter release in the mPFC [15]. Conditioned place preference (CPP) induced by cocaine decreased global DNA methylation in the PFC [16]. However, further studies in animal models are still needed to better understand how excessive and repeated episodes of alcohol consumption alter mPFC function and behavioral control.

DNA methylation is a key epigenetic mechanism in the regulation of gene expression. DNA methylation is catalyzed by DNA methyltransferases, which include DNMT1 and DNMT3A/3B. DNMT1 (maintenance DNMT) has a preference to methylate hemimethylated DNA. Hepatic DNMTase activity was shown to be reduced upon chronic alcohol feeding, which correlated with reduced expression of DNMT1 protein [17]. DNMT3A and DNMT3B regulate de novo methylation. The expression of DNMT3A/3B mRNA was down-regulated in patients with alcohol dependence along with a 10% increase in genome-wide DNA methylation levels [18]. Moreover, systemic inhibition of DNMTs activity decreased excessive alcohol drinking and seeking behaviors in rodents [19]. These studies suggest that DNA methylation may be involved in the mechanisms underlying neural and behavioral responses to alcohol dependence.

Although previous studies have implied a role of DNA methylation in alcohol related-behavior, the mechanisms by which DNA methylation contribute to long-term neuroadaptations in alcohol abuse are presently unknown. Here, using a model of chronic alcohol exposure rats, we first investigated potential roles of DNMTs in regulating the DNA methylation that underlies alcohol effects. Then, we measured the possible contribution of 5-Aza-2’-deoxycytidine (5-Aza-dc, an archetypal DNA methyltransferase inhibitor) on alcohol drinking behavior, locomotor activity and anxiety-like behavior. Finally, we detected the effects of alcohol and 5-Aza-dc, alone and in combination, on Ntf3 and its preferred receptor TrkC expression in the mPFC after long-term alcohol exposure. These results may contribute to the mechanism of DNA methylation on the development of alcohol abuse and provide an initial rationale for exploring the potential of 5-Aza-dc as a treatment for alcohol abuse.

## Materials and methods

### 2.1 Animals

Male Sprague-Dawley rats obtained from the Laboratory Animal Center of Xi’an Jiaotong University were used. Animals were habituated 1 week before experimentation and housed on a 12-h light-dark cycle (light on at 08:00 AM) with access to food and water ad libitum. All animal experiments were approved by the Institutional Animal Care and Use Committees of Xi’an Jiaotong University, and the Animal Research: Reporting of In Vivo Experiments (ARRIVE) guidelines were followed. Rats were aged 8–10 weeks at the beginning of the study.

### 2.2 Experimental procedures

#### 2.2.1 Experiment 1: Chronic alcohol exposure

**Fig 1:**
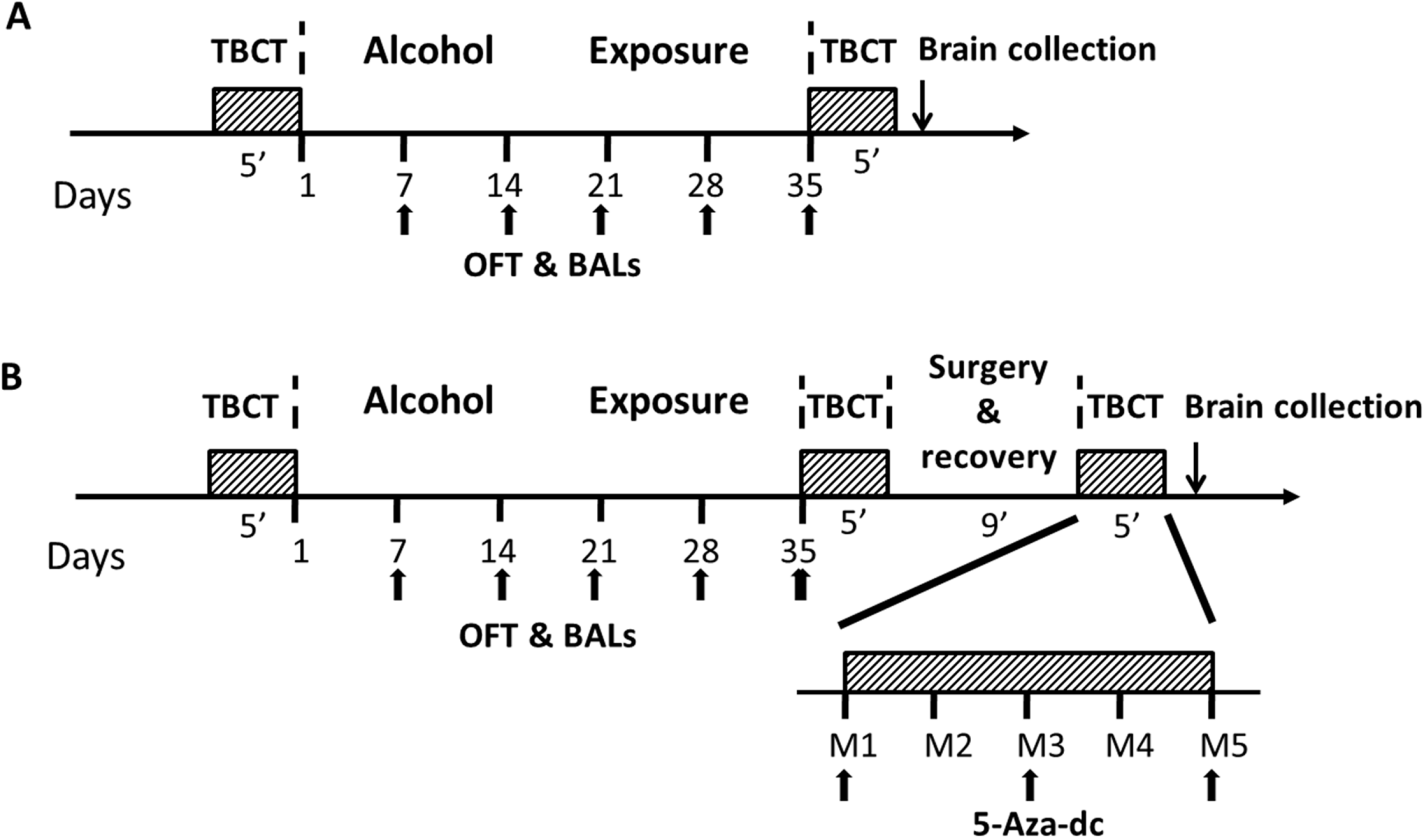
General experimental design. In experiment 1 (A), rats were randomly divided into an alcohol-exposure group (n = 12) and a water-exposure group (n = 12). After baseline drinking, alchol-exposure rats underwent TBCT for 5 consecutive days, and water-exposure rats underwent TBCT at the first and fifth day. Alcohol-exposure group then received alcohol solution as the only liquid source for 5 weeks and water-exposure group received water ad libitum. Next, rats were evaluated for alcohol drinking by TBCT for another 5 days. Then animals were decapitated immediately and brain tissues were collected. In experiment 2 (B), all rats underwent the same protocol as which in experiment 1. Then, rats were randomly divided into four groups, Water+DMSO, Water+5-Aza-dc, Alcohol+DMSO, and Alcohol+5-Aza-dc (n = 8/group) groups, and received mPFC stereotactic surgery followed by recovery for a total of 9 days. Subsequently, rats underwent bilateral intra-mPFC injections of 5-Aza-dc or DMSO at days 1, 3, and 5 followed by 6% (v/v) alcohol or water exposure 30 min later. Alcohol intake and TBCT were monitored every day. After the last TBCT, mPFC were collected.

The general experimental design was performed as described previously [20] with minor modifications (Fig 1A). Rats were assigned randomly to two groups: an alcohol-exposure group (n = 12) and a water-exposure group (n = 12). Anhydrous ethanol (Huada Pharmaceutical Factory, Guangdong, China) was dissolved in sterile water. Alcohol solution was administered at a concentration from 1% to 6% (v/v) for adaptation during the first 6 d. On the next day, alcohol-exposure rats had 24-hour concurrent access to 2 bottles, one with 6% alcohol and another with water for 5 days. Water-exposure rats underwent the two bottle choice test (TBCT) on only the first and fifth days. On each day, the placement of the two bottles was alternated to control for side preference. Alcohol and water consumption were recorded at 5:00 PM every day. Alcohol preference was defined as the volume of alcohol consumption divided by total fluid volume consumed (alcohol + water) ×100%. The volume of alcohol intake was converted to a value in g/kg/d and expressed as the mean±SEM. Alcohol-exposure group then received alcohol solution as the only liquid source for 5 weeks and water-exposure group received water ad libitum. The open field test (OFT) and blood alcohol levels (BALs) were conducted after 7, 14, 21, 28 and 35d of alcohol exposure. Then, rats were evaluated for alcohol drinking by the TBCT for another 5 consecutive days. After the last TBCT, mPFC were collected.

#### 2.2.2 Experiment 2: Intra-mPFC 5-Aza-dc infusion

To examining behavioral and molecular changes after intra-mPFC 5-Aza-dc infusion, rats were randomly divided into four groups depending on the different drug treatments: Water+DMSO, Water+5-Aza-dc, Alcohol+DMSO, Alcohol+5-Aza-dc (n = 8/group; Fig 1B). All rats underwent the same protocol as in *experiment 1*. Then, rats received mPFC stereotactic surgery and recovery for a total of 9 days. Subsequently, rats underwent bilateral mPFC microinjection of 5-Aza-dc or DMSO on days 1 (M1), 3 (M3) and 5 (M5), followed by 6% (v/v) alcohol or water exposure 30 min later. Alcohol intake, TBCT and BALs were monitored every day. OFT were evaluated on the first and last day after the intra-mPFC injection of 5-Aza-dc.

### 2.3 Open field test (OFT)

A black rectangular box was used (100 cm×100 cm×45 cm) for the OFT. The box was illuminated with three 30 W fluorescent bulbs placed 2 m above the box. The experiment was carried out in a sound-attenuating room at 8:00. Rats (n = 12/group) were placed in the corner of the box and allowed 15 min of exploration. The total distance travelled and the percent of time in the center were analyzed by a computerized video tracking system (SMART, Panlab SL, Barcelona, Spain).

### 2.4 Blood alcohol levels (BALs)

After the OFT, blood samples (0.4 ml) of alcohol-exposure rats (n = 12/group) were taken via tail vein. 0.1ml of plasma was used to determine BALs using an EnzyChromTM Ethanol Assay Kit (ECET- 100, BioAssay Systems, Hayward, CA, USA).

### 2.5 Surgery

Rats (n = 8/group) were anesthetized with sodium pentobarbital (65 mg/kg, i.p.) and secured in a stereotaxic apparatus (RWD, Shenzhen, China). Under aseptic conditions, stainless steel guide cannulas (26 gauge; Plastics One) were implanted bilaterally 1 mm above the region of interest (mPFC stereotaxic coordinates: anteroposterior, +3.2 mm from bregma, ±0.7 mm lateral from midline and −4.0 mm from skull) [21]. A stainless steel obturator of the same length was placed in each cannula to ensure clearance. Animals were allowed to recover for 7 days, during which they received sodium penicillin (0.2 million U/day for 5 days, i.p.) to prevent intracerebral infection. Following recovery, animals were habituated to dummy cannula removal before drug infusions.

### 2.6 Drug and microinjection

The DNA methyltransferase inhibitor 5-Aza-dc (Sigma, USA) was diluted with 0.2% dimethylsulfoxide (DMSO, diluted with saline) at the concentration 2.0μg/μl [22, 23]. The vehicle groups received isovolumetric 0.2% DMSO injections. Microinjections were performed using a 1.0-μl Hamilton syringe connected to a 30-gauge injector that terminated 1 mm below the tip of the guide cannula. 5-Aza-dc (0.5μl/side) or DMSO was infused into the mPFC bilaterally over 2 min. The injection cannulas were left in place for an additional 2 min to allow for drug diffusion and to prevent backflow. Then, the obturators were reinserted into the guide cannula.

### 2.7 RNA isolation, reverse transcription and qPCR

After the last TBCT, animals were decapitated immediately both in Experiment 1 and Experiment 2 (Fig 1). Bilateral samples from the mPFC were dissected out. Half of mPFC were stored at -80°C and used to extract the protein, and the other half were placed in Sample Protector for RNA (TaKaRa, Japan) and used for RNA isolation. Total RNA was extracted using TaKaRa MiniBEST Universal RNA Extraction Kit following the manufacturer’s instructions. cDNA was reverse transcribed from total RNA, and 5 ng cDNA was amplified using an iQ5 Real-Time PCR Detection System (Bio-Rad, Hercules, CA). The primers (AuGCt, China) used to analyze gene expression were as follows: DNMT1 gene, forward primer 5' GTTCCTTGTAGGCGA GTGTG 3' and reverse primer 5' TTGCGTAGTCCTGGCTGTAC 3'; DNMT3A gene, forward primer 5' GCAAAGTGAGGACCATTACC 3', reverse primer 5' GCCAAACACCCTTTCCAT 3'; DNMT3B gene, forward primer 5' GAATTTGAGCAGCCCAGGTTG 3', reverse primer 5' TGAGAAGAGCCTTCC TGTGCC 3'; Ntf3 gene, forward primer 5' GGCAACAGAGACGCTACAAT 3', reverse primer 5' TCCTCCGTGGTGATGTTCTA 3'; TrkC gene, forward primer 5' ACCATGGCATCACTACACCTTC 3',reverse primer 5' CTTAGATTGTAGCACTCAG 3'; GAPDH gene, forward primer5' ATGGGGAAGGTGAAGGTCG 3', reverse primer 5' GGGGTCATTGATGGCAACAA 3'. PCR was performed in a 10μl reaction volume. cDNA concentrations were calculated according to the ΔΔCt method, corrected for differences in PCR efficiency, and normalized to glyceraldehyde-3-phosphate dehydrogenase (GAPDH).

### 2.8 Western blotting

Brain tissues were lysed in a pre-cooled RIPA buffer (50 mM Tris-HCl pH 7.4, 50 mM NaCl, 1% Triton X-100, 2 mg/ml protease inhibitors and 2 mg/ml phosphatase inhibitors), homogenized with ultrasound homogenizer, incubated on ice for 30 min and centrifuged at 12,000⨯*g* for 15 min at 4°C. The protein content was determined using the bicinchoninic acid (BCA) method (PPLYGEN, Beijing, China). The protein samples were subjected to various concentrations of SDS-PAGE according to the different molecular weights (DNMT1 8%, DNMT3A, DNMT3B and TrkC 10%, Ntf3 12%) and transferred to PVDF membranes. The membranes were blocked with 5%(w/w) non-fat dried milk in Tris-buffered saline (TBS) (500 mM NaCl, 20 mM Tris-HCl pH 7.4) containing 0.05% Tween-20 for 1.5 h and incubated overnight with one of the following antibodies at 4°C: anti-DNMT1 polyclonal antibody (1:1000; Epigentek), anti-DNMT3A polyclonal antibody (1:400; Epigentek), anti-DNMT3B polyclonal antibody (1:500; Epigentek), anti-Ntf3 polyclonal antibody (1:1000; Abcam), anti-TrkC monoclonal antibody (1:1000, Cell Signaling Technology). The next day, the membranes were washed three times with 0.1% Tween-20 TBS (pH 7.4) and incubated with horseradish peroxidaseconjugated anti-rabbit or anti-mouse secondary antibodies for 1.5 h. An enhanced chemiluminescence kit (Millipore, MA, USA) was used to detect immunoreactive protein bands. The band intensities were analyzed using the Quantity One software (Bio-Rad, Hercules, USA) to calculate the target protein vs. the inner control (β-actin 1:1000) for each protein. In order to eliminate the interference of the background signal, normalisation was carried out with reference to the total lane protein as determined using the stain-free technology by Bio-Rad.

### 2.9 Cannula verification

At the end of experiments, rats were anesthetized with sodium pentobarbital (65mg/kg) and transcardially perfused. Cannula placements were assessed using Nissl staining with a section thickness of 30 μm under light microscopy. Rats with misplaced cannulas were excluded from statistical analysis.

### 2.10 Statistics

All data were expressed as mean ± S.E.M. Repeated measures (RM) one-way analysis of variance (ANOVA) was used to assess the alcohol intake, BALs and alcohol preference in Experiment 1. The Sidak’s post hoc test was used when necessary. Body weight, the open-filed behaviors in Experiment 1, and alcohol intake, total fluid intake, alcohol preference, BALs, the open-field behaviors in Experiment 2 were analyzed by using RM two-way ANOVA followed by Sidak’s post hoc test. The mRNA and protein levels of DNMTs in Experiment 1 were determined by unpaired t-test. The mRNA and protein levels of DNMTs, Ntf3, TrkC in Experiment 2 were determined by two-way ANOVA followed by Newman–Keuls post hoc test. The accepted level of significance for all tests was p < 0.05.

## Results

### 3.1 Body weight, alcohol intake, BALs, TBCT and OFT after chronic alcohol exposure

**Fig 2:**
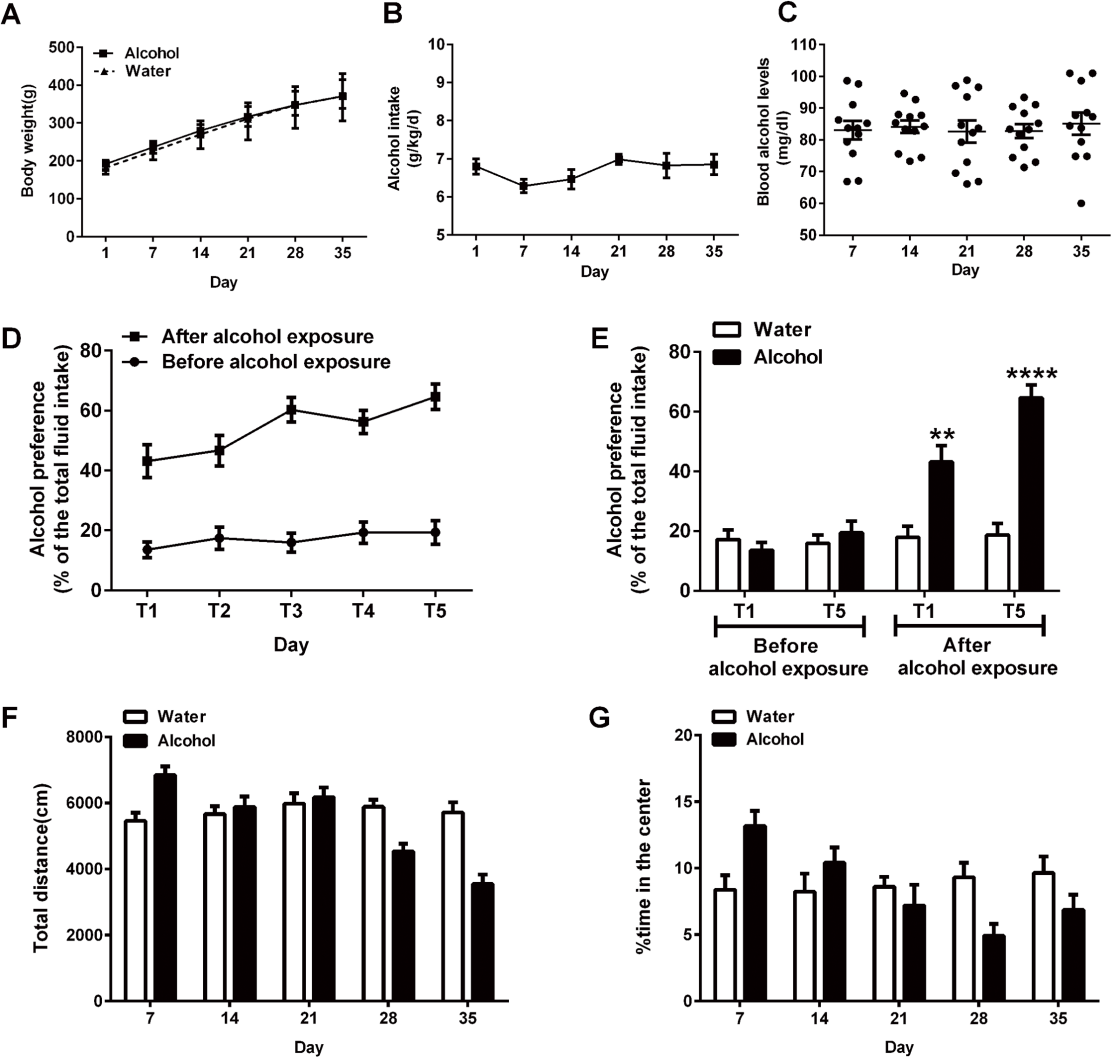
Effects of alcohol exposure on body weight, alcohol intake, BALs, TBCT and OFT. (A) Changes in body weight during the alcohol exposure period (n = 12 per group). (B) Daily alcohol intake (g of pure alcohol per kg of body weight) during the 35-day alcohol exposure period (n = 12). (C) BALs (mg/dl, n = 12) during the alcohol exposure period. (D) Alcohol preference (% of total fluid intake) in alcohol-exposure rats for 5 days before and after alcohol exposure (n = 12/group). (E) The alcohol preference of the water- and alcohol-exposure rats at 1d and 5d of the TBCT before and after alcohol exposure (n = 12/group). The total distance travelled (F) and %time in the center (G) (n = 12/group).The values represent the mean ± SEM. ** p < 0.01, **** p < 0.0001 vs. water-exposure controls.

RM two-way ANOVA revealed no significant difference in weight gain between the water- and alcohol-exposure groups [F _Alcohol (1, 22)_ = 0.649, p = 0.429]. All rats exhibited an increase in body weight during the alcohol- or water-exposure period [F _Time (5, 110)_ = 932.3, p < 0.0001] (Fig 2A). Rats stably consumed an average of 6.47± 1.08 g/kg/day of alcohol for 35 d [F_(5, 66)_ = 2.268, p = 0.121] (Fig 2B), which resulted in an average BALs of (83.54±1.24) mg/dl. RM one-way ANOVA revealed that the BALs did not differ significantly over the alcohol exposure period [F _(4, 55)_ = 0.118, p = 0.936] (Fig 2C).

Before chronic alcohol exposure, RM one-way ANOVA showed a stable baseline alcohol preference in the alcohol-exposure groups [F_(4, 55)_ = 0.467, p = 0.721]. The average alcohol preference of the alcohol groups was 17.12±1.50%. After alcohol exposure for 35 days, the TBCT was performed again. RM one-way ANOVA revealed an effect of time on alcohol preference [F_(4, 55)_ = 3.905, p < 0.05] in alcohol-exposure rats. The post hoc test showed alcohol preference was increased (p < 0.05) at 5d than which at 1d. In spite of this, the minimum alcohol preference in alcohol-exposure rats (43.10±5.54%) was much higher than the average value before alcohol exposure (Fig 2D). Before alcohol exposure, there was no significant difference in alcohol preference between the water- and alcohol-exposure groups at 1d and 5d of the TBCT [1d: t_(1, 22)_ = 0.870, p = 0.394; 5d: t_(1, 22)_ = 0.731, p = 0.473] (Fig 2E). However, after long-term alcohol exposure, the alcohol-exposure groups had a significantly increased alcohol preference than the water-exposure groups [1d: t_(1, 22)_ = 3.786, p < 0.01; 5d: t_(1, 22)_ = 7.994, p < 0.0001] (Fig 2E).

The open-field behaviors were analyzed at 7, 14, 21, 28 and 35 days after chronic alcohol exposure. As shown in Fig 2F, two-way ANOVA was conducted with treatment (alcohol or water) as the between-subjects factor and time as the within-subjects factor. ANOVA revealed a significant main effect of time [F _(4, 88)_ = 24.150, p < 0.0001] but no significant effect of treatment [F _(1, 22)_ = 2.868, p = 0.113]. Compared with the water-exposure groups, there was an increasing trend (WE:5457.75±192.75 cm VS AE:7017.95±213.73 cm) in the total distance travelled of alcohol-exposure rats at 7 d, which indicated that short-term alcohol exposure could induce locomotor stimulatory effects. However, at 14 d and 21 d, the total distance in the water- and alcohol-exposure rats was similar, suggesting adaptation of the nervous system. At 28 d and 35 d, a decreasing trend (WE:5824.94±144.86cm VS AE:4494.54±180.74 cm at 28d; WE:5602.01±222.11cm VS AE:3635.22±208.12cm at 35d) in the total distance was detected in alcohol-exposure rats, which reflected the inhibitory effects of chronic alcohol exposure. RM two-way ANOVA for % time in the center revealed a significant time effect [F _(4, 88)_ = 4.607, p < 0.05] but no treatment effect [F _(1, 22)_ = 0.105, p = 0.751] (Fig 2G). The exploration time in the center of the alcohol-exposure rats had an increasing trend than water-exposure rats at 7d and 14 d (WE:8.38±1.10 VS AE:13.18±1.13 at 7d; WE:8.24±1.35 VS AE:10.40±1.16 at 14d), which was similar between two groups at 21d. At 28d and 35d, there was a decreasing trend in alcohol-exposure rats (WE:9.32±1.10 VS AE:4.93±0.89 at 28d; WE:9.64±1.25 VS AE:6.87±1.13 at 35d), suggesting anxiogenic effects of long-term alcohol exposure.

### 3.2 The mRNA and protein levels of DNMTs after chronic alcohol exposure

**Fig 3:**
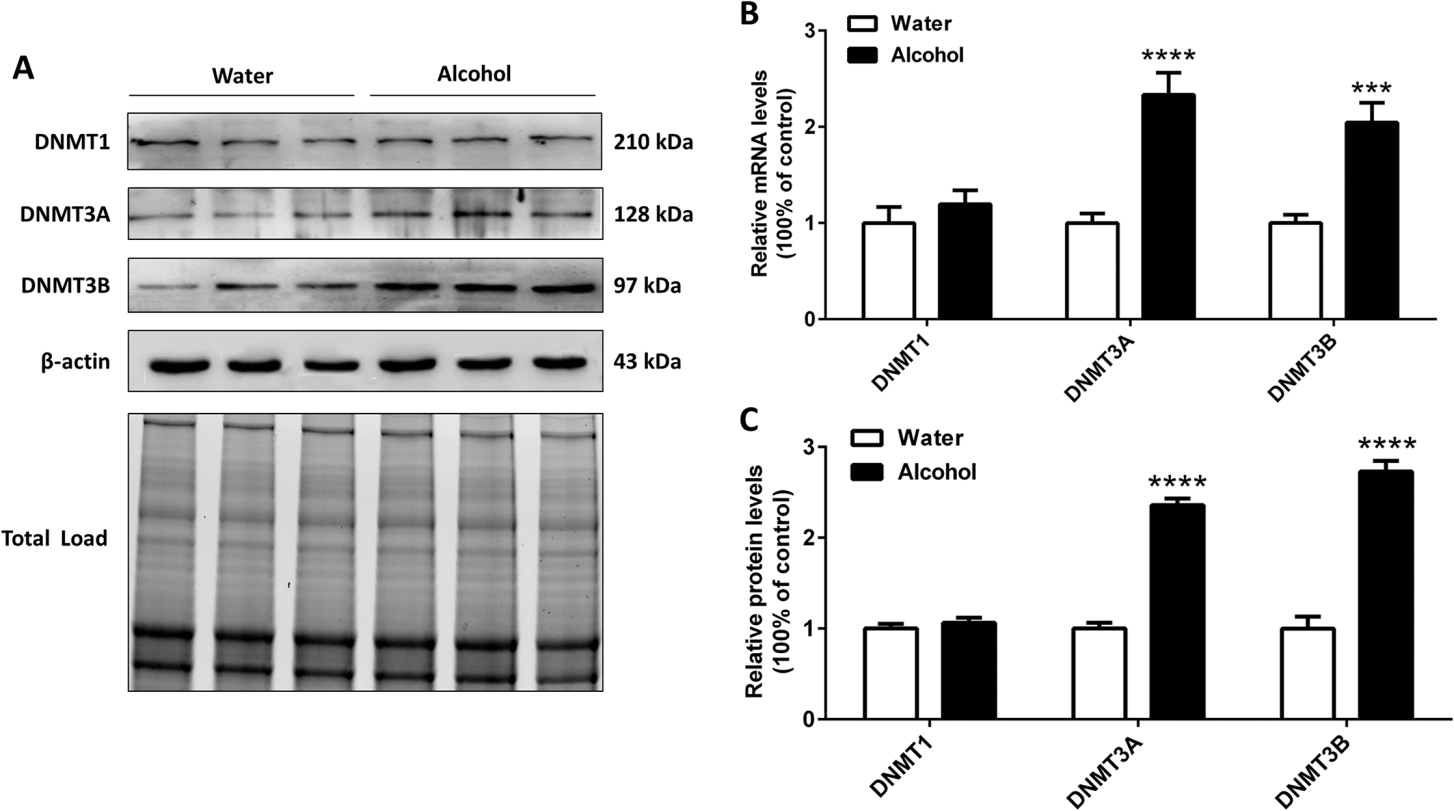
(A) Representative immunoblots and total protein image. Effects of alcohol exposure on the mRNA (B) and protein (C) expressions of DNMT1, DNMT3A and DNMT3B in the mPFC (n = 12/group). The data represent the mean ± SEM. *** p < 0.001, **** p < 0.0001 vs. water-exposure controls.

After 35 days of alcohol exposure, the mRNA and protein levels of DNMTs in the mPFC were measured. As shown in Fig 3B, the mRNA levels of DNMT1 were not significantly changed [t_(1, 22)_ = 0.885, p = 0.391]. There was a significant increase in the mRNA expression of DNMT3A [t_(1, 22)_ = 5.367, p < 0.0001] and DNMT3B [t_(1, 22)_ = 4.652, p < 0.001] in the alcohol groups compared with the water groups. As shown in Fig 3C, consistent with the results of mRNA levels, DNMT1 protein level was not significantly changed [t_(1, 22)_ = 0.824, p = 0.424]. The protein levels of DNMT3A [t_(1, 22)_ = 13.94, p < 0.0001] and DNMT3B [t_(1, 22)_ = 9.8, p < 0.0001] were upregulated following chronic alcohol exposure.

### 3.3 Intra-mPFC DNMTs inhibitor (5-Aza-dc) infusions modulate alcohol-related behaviors

**Fig 4:**
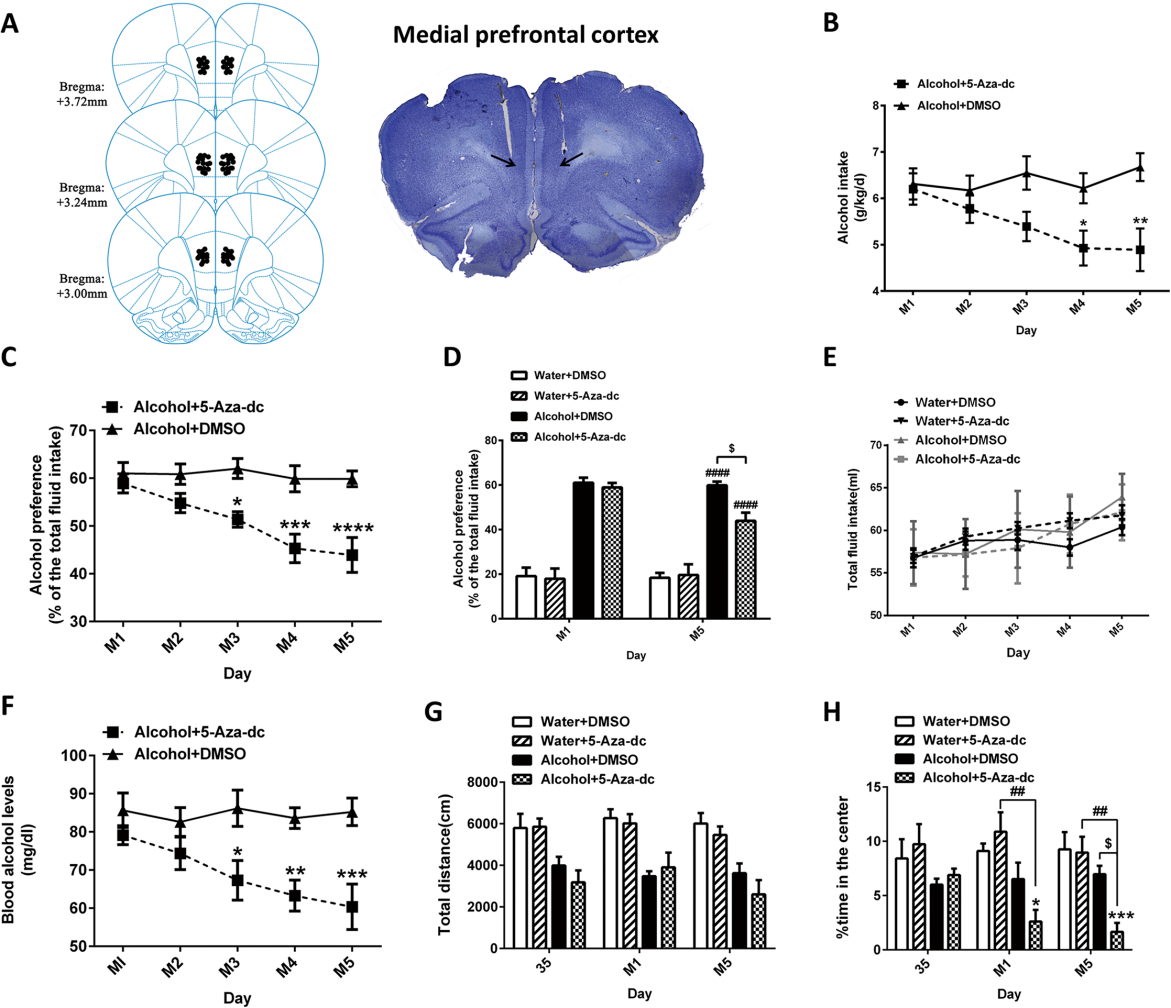
(A) Schematic illustrations and representative photomicrographs of the intracranial cannula infusion sites in the mPFC. 5-Aza-dc infusion decreased alcohol intake (B) and alcohol preference (C) in alcohol-exposure rats, corresponding to the reduced blood alcohol levels (F). (D) The alcohol preference in water- and alcohol-exposure rats at 1d and 5d after 5-Aza-dc treatment. (E) 5-Aza-dc did not affect the total fluid intake. * p < 0.05, ** p < 0.01, *** p < 0.001, **** p < 0.0001 and $ p < 0.05 indicate differences from the Alcohol+DMSO group. #### p < 0.0001, differences compared to Water+DMSO group. (G) 5-Aza-dc had no effect on the locomotor activity of alcohol-exposure rats. (H) 5-Aza-dc decreased the exploration time in the center in alcohol-exposure rats. * p < 0.05, *** p < 0.001, differences compared to the Water+DMSO group; $ p < 0.05, differences compared to the Alcohol+DMSO group; ## p < 0.01, differences compared to Water+5-Aza-dc group; n = 8/group.

To investigate the role of 5-Aza-dc in alcohol-related behaviors, rats received intra-mPFC 5-Aza-dc infusions. Schematic illustrations and representative photomicrographs of the mPFC cannulae fusion sites were presented in Fig 4A. For alcohol intake (Fig 4B), RM two-way ANOVA, with treatment (5-Aza-dc or DMSO) as the between-subjects factor and time (M1, M2, M3, M4 and M5) as the within-subjects factor, showed significant effect of treatment [F _(1, 14)_ = 9.707, p < 0.01] and treatment×time interaction [F _(4, 56)_ = 2.540, p < 0.05]. The post hoc analyses showed a significant decrease in alcohol intake in Alcohol+5-Aza-dc rats than in Alcohol+DMSO rats at M4 (p = 0.048) and M5 day (p = 0.003). Alcohol+DMSO rats had stable alcohol consumption (6.38±0.14 g/kg/d). In addition, 5-Aza-dc treatment significantly lowered the alcohol preference of the alcohol-exposure rats (Fig 4C). Two-way ANOVA was performed with treatment as the between-subjects factor and time as the within-subjects factor. ANOVA revealed a significant effect of treatment [F _(1, 14)_ = 17.430, p < 0.001] and time [F _(4, 56)_ = 6.588, p < 0.001] with a significant interaction [F _(4, 56)_ = 4.635, p < 0.01]. The post hoc tests showed a significant decreased alcohol preference in Alcohol+5-Aza-dc rats than in Alcohol+DMSO rats (p < 0.05 at M3, p < 0.001 at M4 and p < 0.0001 at M5). Meanwhile, the alcohol preference of all groups on M1 and M5 was evaluated (Fig 4D). At M1, two-way ANOVA revealed a significant effect of alcohol [F _(1, 28)_ = 153.1, p < 0.0001] but not 5-Aza-dc. At M5, there were significant effects of both alcohol [F _(1, 28)_ = 98.69, p < 0.0001] and 5-Aza-dc [F _(1, 28)_ = 4.901, p < 0.05], with a significant interaction between them [F _(1, 28)_ = 6.757, p < 0.05]. The post hoc tests showed a significant increase in alcohol preference in alcohol-exposure rats compared with water-exposure rats (p < 0.0001) and a significant decrease in alcohol preference in Alcohol+5-Aza-dc rats compared with Alcohol+DMSO rats (p < 0.05). During the 5 days of intra-mPFC 5-Aza-dc infusions, neither the administration of alcohol nor 5-Aza-dc affected the total fluid intake (p > 0.05) (Fig 4E). To determine if BALs affected the effects of 5-Aza-dc on alcohol-related behavior, blood samples were taken after the TBCT, ANOVA revealed a significant effect of treatment [F _(1, 14)_ = 35.160, p < 0.0001] and treatment×time interaction [F _(4, 56)_ = 2.760, p < 0.05]. The post hoc tests showed significant decreased BALs in Alcohol+5-Aza-dc rats than in Alcohol+DMSO rats (p < 0.05 at M3, p < 0.01 at M4 and p < 0.001 at M5) (Fig 4F).

The open-field behavior was examined before and after 5-Aza-dc treatment. For total distance travelled, as shown in Fig 4G, chronic alcohol exposure for 35 days, consistent with the results of Experiment 1, the locomotor activity of alcohol-exposure rats increased initially and then decreased to the similar level as water-exposure rats, finally it showed a decreasing trend (not fully shown in the figure). After 5-Aza-dc treatment, at M1, two-way ANOVA revealed a significant alcohol effect [F _(1, 28)_ = 25.46, p < 0.0001] but no 5-Aza-dc effect [F _(1, 28)_ = 0.031, p = 0.862]. Similarly, at M5, there was a significant effect of alcohol [F _(1, 28)_ = 25.470, p < 0.0001] but no significant effect of 5-Aza-dc [F _(1, 28)_ = 2.257, p = 0.146]. These results suggested that 5-Aza-dc has no effect on alcohol-induced locomotor activity of rats. In addition, we found 5-Aza-dc potentiate the anxiogenic effects of alcohol-exposure rats (Fig 4H). At the 35d of alcohol exposure, a decreasing trend in exploration time in the center was found for alcohol-exposure rats than water-exposure rats. At the 1d of 5-Aza-dc treatment, two-way ANOVA revealed a significant effect of alcohol [F _(1, 28)_ = 15.650, p < 0.001] and alcohol×5-Aza-dc interaction [F _(1, 28)_ = 4.286, p < 0.05], but there was no significant effect of 5-Aza-dc [F _(1, 28)_ = 0.608, p = 0.442]. The post hoc analysis confirmed that the exploration time in the center of the Alcohol+5-Aza-dc rats significantly decreased (p < 0.05, compared to the Water+DMSO groups; p < 0.01, compared to the Water+5-Aza-dc groups). At M5, two-way ANOVA revealed significant effects of alcohol [F _(1, 28)_ = 15.490, p < 0.001] and 5-Aza-dc [F _(1, 28)_ = 5.313, p < 0.05], as well as an interaction between them [F _(1, 28)_ = 4.214, p < 0.05]. The post hoc analysis showed that 5-Aza-dc decreased the exploration time in the center of alcohol-exposure rats (Water+DMSO vs. Alcohol+5-Aza-dc p < 0.001; Water+5-Aza-dc vs. Alcohol+5-Aza-dc p < 0.01; Alcohol+DNMSO vs. Alcohol+5-Aza-dc p < 0.05).

### 3.4 Intra-mPFC DNMTs inhibitor (5-Aza-dc) infusions modulated the expression of DNMTs associated with the changed Ntf3-TrkC signaling pathway

**Fig 5:**
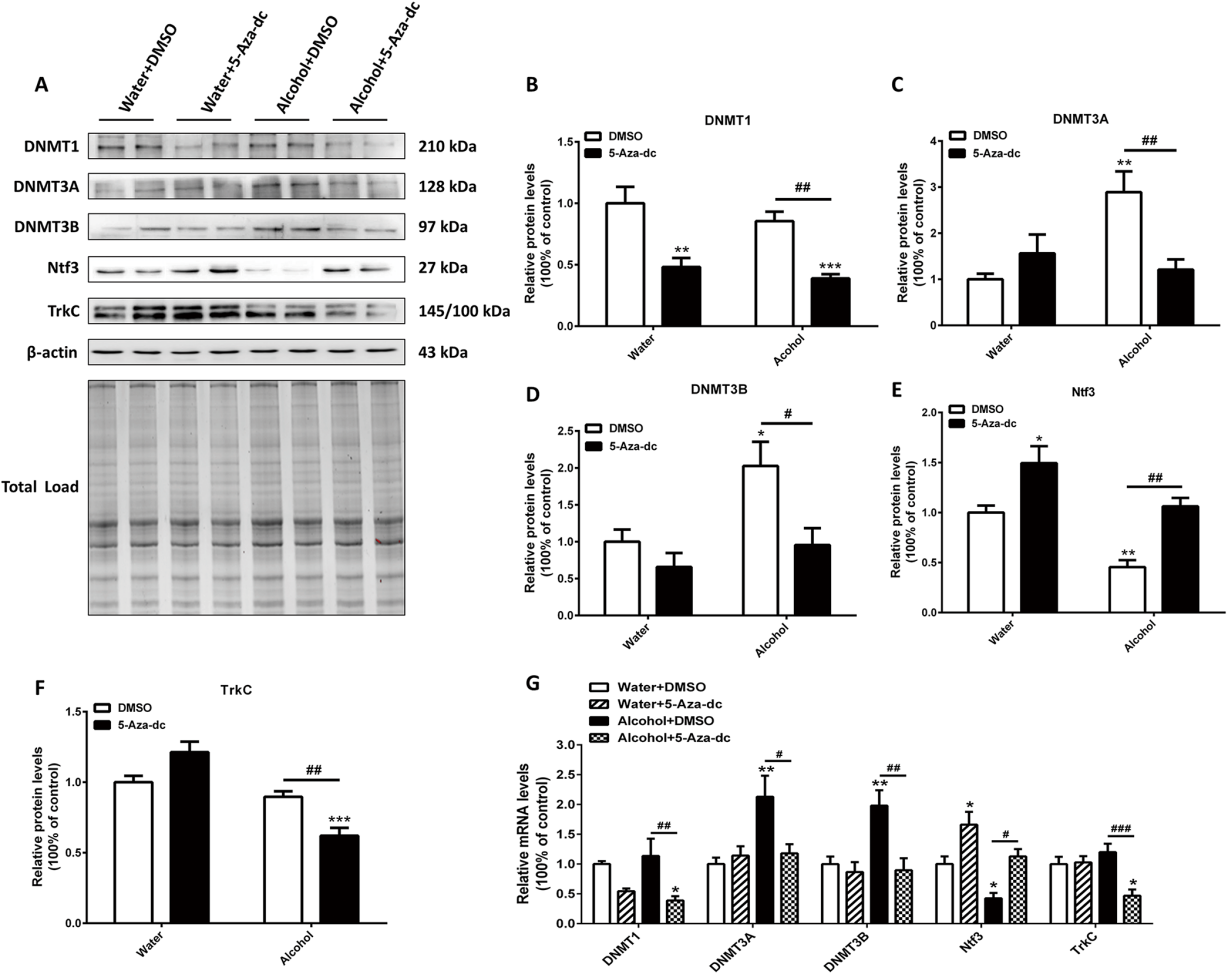
Effects of 5-Aza-dc and alcohol on DNMTs and Ntf3/TrkC expressions in the mPFC. Fig 5A shows representative immunoblots and total protein image. 5-Aza-dc decreased the protein level of DNMT1 (B), whereas it reversed the alcohol-induced overexpression of DNMT3A (C) and DNMT3B (D). 5-Aza-dc prevented the Ntf3 reduction induced by chronic alcohol exposure (E) but had no effect on TrkC (F). (G) Differential expression of mRNA for DNMTs, Ntf3 and TrkC. * p < 0.05, ** p < 0.01, *** p < 0.001, differences compared to the Water+DMSO group; # p < 0.05, ## p < 0.01, ### p < 0.01, differences compared to the other corresponding groups; n = 8/group.

To understand the mechanisms of 5-Aza-dc treatment underlying the behavioral response to alcohol, we compared the protein levels of DNMTs in rats with 5-Aza-dc treatment to the ones without 5-Aza-dc treatment. Representative blots and total protein blots are showed in Fig 5A. As shown in Fig 5B, two-way ANOVA revealed significant effects of 5-Aza-dc [F _(1, 28)_ = 31.77, p < 0.0001] but not alcohol [F _(1, 28)_ = 1.855, p = 0.184] on DNMT1 expression. For DNMT3A expression (Fig 5C), two-way ANOVA revealed significant effects of alcohol [F _(1, 28)_ = 5.853, p < 0.05] and a significant interaction between alcohol and 5-Aza-dc [F _(1, 28)_ = 12.47, p < 0.01]. The post hoc analysis showed that alcohol significantly increased the levels of DNMT3A (2.89-fold in the Alcohol+DMSO group, p < 0.01). Compared with Alcohol+DMSO groups, 5-Aza-dc reversed the high levels of DNMT3A (2.39-fold in the alcohol+5-Aza-dc group, p < 0.05). There were significant effects of both alcohol [F _(1, 28)_ = 7.242, p < 0.05] and 5-Aza-dc [F _(1, 28)_ = 8.262, p < 0.01] on DNMT3B expression, with no obvious interaction between them (Fig 5D). Alcohol significantly increased the levelof DNMT3B (2.03-fold in the Alcohol+DMSO group, p < 0.05). Compared with Alcohol+DMSO groups, 5-Aza-dc reversed the high alcohol-induced levels of DNMT3B (2.12-fold in the Alcohol+5-Aza-dc group, p < 0.05). Whereas β-actin levels were similar across all groups, indicating equivalent protein contents in samples.

To gain further insight into DNA methylation and related molecular alteration in the mPFC, we analyzed the potential target genes expression (Ntf3 and its preferred receptor TrkC), which have emerged as potent mediators of neuron development and synaptic plasticity. For Ntf3 expression (Fig 5E), two-way ANOVA revealed significant effects of alcohol [F _(1, 28)_ = 21.10, p < 0.0001] and 5-Aza-dc [F _(1, 28)_ = 26.78, p < 0.0001] but failed to show a significant interaction. The post hoc analysis revealed that Ntf3 expression (Water+DMSO vs. Alcohol+DMSO p < 0.01) was downregulated following chronic alcohol exposure. The downregulation of Ntf3 was reversed by 5-Aza-dc administration (Alcohol+DMSO vs. Alcohol+5-Aza-dc p < 0.01). For TrkC (Fig 5F), two-way ANOVA showed significant effects of alcohol × 5-Aza-dc [F _(1, 28)_ = 19.44, p < 0.001]. Compared with the Alcohol+DMSO groups, the Alcohol+5-Aza-dc groups had a decreased expression of TrkC (1.44-fold, p < 0.01). DNMTs, Ntf3 and TrkC mRNA levels in the mPFC were in agreement with the protein expression results (Fig 5G).

## Discussion

In the present study, we discovered three main findings. First, it was found that chronic alcohol exposure had a significant effect on the expression of DNA methyltransferase in the mPFC. Chronic alcohol exposure increased the mRNA and protein expression of DNMT3A and DNMT3B. However, 5-Aza-dc could reverse the higher levels of DNMT3A and DNMT3B. Second, 5-Aza-dc reduced both alcohol consumption and preference in alcohol-exposure rats, corresponding to the reduced blood alcohol levels. Interestingly, 5-Aza-dc tended to promote anxiety-like behaviors but had no effect on locomotor activity in alcohol exposure rats. Third, 5-Aza-dc attenuated alcohol-induced downregulation of Ntf3. In accordance with the status of Ntf3, the expression of TrkC in the same signaling pathway changed accordingly.

5-Aza-dc is a deoxycytidine analog that is incorporated into DNA, which prevents the resolution of a covalent reaction intermediate [24] and leads to DNA methyltransferase being inactivated in the form of a covalent protein-DNA adduct. As a result, cellular DNA methyltransferase is rapidly depleted, and concomitantly genomic DNA is demethylated as a result of continued DNA replication [25]. 5-Aza-dc is well known to be cytotoxic and its toxicity depends on the dosage and exposure time [26]. Relevant research has shown that in the concentration range of 0.1ug/ulto 2ug /ul in specific brain region [22, 23, 27, 28], 5-Aza-dc did not interfere with the locomotor activities of rats, in line with our OFT results. In addition, our results suggest that 5-Aza-dc (1μg /0.5μl/side) in the mPFC do not affect the total fluid intake (Fig 4E) or body weight (S1 Fig) of rats. However, the optimal dose-schedule for 5-Aza-dc with a novel mechanism of action remains to be determined [29], so in subsequent studies, it is necessary to directly test whether 5-Aza-dc may cause a chemical lesion in nerve cells. To elucidate whether 5-Aza-dc in the mPFC affected alcohol intake and alcohol preference, rats were given repeated 5-Aza-dc microinjections into the mPFC after chronic alcohol exposure. Our results showed that intra-mPFC injection of 5-Aza-dc do not influence the alcohol preference of water-exposure rats. However, 5-Aza-dc significantly decreased alcohol intake and alcohol preference of alcohol-exposure rats, indicating that methyltransferase inhibition in the mPFC could impair alcohol drinking behavior, which was consistent with a previous study that reported systemic administration of methyltransferase inhibitor reduced binge-like alcohol drinking in mice [19]. In addition, injections of the DNMTs inhibitor RG108 into the mPFC prevented escalation of alcohol consumption, dependence-induced downregulation synaptotagmin 2 (Syt2) gene expression and hypermethylation on CpG#5 of its first exon [15].

The OFT is commonly used to assess the sedative, toxic, or stimulant effects of compounds [30, 31]. The duration of time spent and the measured activity in the central zone likely gage some aspects of emotionality-like behavior, including anxiety and exploratory drive [32–34]. In the present study, alcohol exerted locomotor stimulant and anxiolytic effects in rats following 7 days of alcohol exposure, suggests that short-term exposure to low-dose alcohol tends to increase locomotor activity and has, to some extent, anti-anxiety effects. However, these behavioral differences between alcohol and water groups diminished in 14d and 21d. This apparent discrepancy may due to an adaptation of the nervous system to the alcohol. Strikingly, after 28 d of alcohol exposure, rats showed a decreasing trend on total distance and exploration time in the center, reflecting the inhibitory effects of long-term alcohol exposure. Similar to our findings, Boerngen-Lacerda indicated that the increase in locomotor activity was only observed after 7 and 14 days of ethanol treatment in the OFT; however, ethanol increased the open-arm time in the plus-maze, which exerted an anxiolytic effect, including after acute and chronic administration of alcohol [35]. Because alcohol abuse is an extremely complex progress, different methods have been employed to establish models of alcohol dependence, and therefore, these inconsistent results are understandable.

In our study, 5-Aza-dc did not interfere with the locomotor activities of rats. In agreement with our findings, Sales et al. noted that systemic administration or intra-hippocampal injection of DNMT inhibitors did not induce any locomotor effects in the OFT of rats [36]. Xing et al. found that bilateral intra-VLO (ventrolateral orbital cortex) injections of 5-Aza-dc did not cause general impairments in locomotor activity [28]. Recent studies have examined the effect of DNA methyltransferase in regulating depression, stress, and anxiety-like behavior. Interestingly, we found that in alcohol-drinking rats, 5-Aza-dc decreased their exploration time in the center area, which suggests that 5-Aza-dc could potentiate anxiety-like behaviors. In a previous study, knocking down mPFC-DNMT3A in naive mice induced anxiety-like behavior while overexpressing this gene had an anxiolytic effect [37]. Additionally, systemic and hippocampal inhibition of DNA methylation induced antidepressant-like effects in rats [36], and 5-Aza-dc could potentiate the behavioral effects of antidepressant drugs in the forced swimming test (FST) [38]. Moreover, both rodent models and human studies have demonstrated that DNA methylation is involved in memory and emotion [39–41]. With the above research, our data further support the hypothesis that DNMT3A and DNMT3B are important regulators involved in the development of anxiety-like behavior.

Neurotrophins have been proposed as key regulators of long-term synaptic modifications related to learning and memory maintenance[42]. In particular, Ntf3 and its preferred receptor TrkC have been shown to enhance axonal outgrowth and regulate structural synaptic plasticity in a number of cell types [43–45]. Repeated intra-VTA Ntf3 contributed to the initiation of behavioral sensitization to cocaine by activating the Ras/MAPK signal transduction system in rats [46]. Mice with a central nervous system-wide deletion of Ntf3 had attenuated opiate withdrawal reactions which were restored by transgene-derived overexpression of Ntf3 in noradrenergic neurons [47]. When rats received chronic ethanol treatment during gestation, the neurotrophin receptors TrkA, TrkB, and TrkC levels in the hippocampus, septum, and cerebellum were significantly decreased in P10 fetal rats[48]. However, evidence of the effects of Ntf3/TrkC on alcohol dependence is scarce. In our study, 5-Aza-dc micro-infusion into the mPFC reversed alcohol-induced decreased Ntf3 protein levels by decreasing the levels of DNMT3A and DNMT3B. These results suggested that Ntf3 seems to be directly regulated by DNA Methyltransferase, although the methylation level of the CpG sites in the Ntf3 promoter region was not significantly different (S1 Table). Maybe non-CpG methylation or gene body methylation played a crucial role in the development of alcohol abuse [49]. Interestingly, 5-Aza-dc also increased the protein level of water-exposure rats, suggesting that the Ntf3 expression doesn’t appear to depend on the combination of administration and 5-Aza-dc administration. This phenomenon may be because in the normal nerve cells, 5-Aza-dc also captures DNA methyltransferase, resulting in the demethylation of Ntf3, and induces its gene expression. Strangely, alcohol or 5-Aza-dc alone had no effects on TrkC, whereas alcohol and 5-Aza-dc co-treatment decreased the expression of TrkC. On one hand, it is possible that the increased Ntf3 levels could passivate TrkC, allowing TrkC to adapt to the combined effects of alcohol and 5-Aza-dc on Ntf3. On the other hand, though Ntf3 binds to TrkC with high affinity, it also binds to both TrkA and TrkB with lower affinity [42]. We cannot exclude that these receptors played a role in the change of Ntf3. Moreover, the reported effects of alcohol exposure on Trk receptors are complex and differ depending on the postnatal age of measurement, brain region and sex of the animal [50, 51], making it difficult to generalize about possible relationships between neurotrophin levels and receptor function. Nevertheless, it is reasonable to assume that Ntf3 is a potential target of epigenetic modifications involved in alcohol-drinking behaviors.

In summary, our results demonstrated that 5-Aza-dc decreased both alcohol consumption and alcohol preference in the chronic alcohol-exposure rats; meanwhile, it prevented alcohol-induced Ntf3 reduction in the mPFC, probably by inhibition of DNMT3A and DNMT3B. Thus, appropriate doses of DNMT inhibitors may have a pharmacy therapeutic potential for alcohol abuse.

## Supporting information

S1 Fig The effects of 5-Aza-dc treatment on the body weight of four groups (n = 8/group). (TIF)

S1 Methods Bisulfite direct sequencing, quantitation of methylation levels and statistical analysis. (DOCX)

S1 Table Methylation status of Ntf3 promoter region in the mPFC of rats (n = 7~8/group). 0 = no methylation, 1 = <33% methylation.
(DOCX)
