# Supplementary material for: 5-Aza-2’-deoxycytidine in the medial prefrontal cortex regulates alcohol-related behavior and Ntf3-TrkC expression in rats
Source: PLoS One. 2017 Jun 14;12(6):e0179469. doi: 10.1371/journal.pone.0179469 (PMC5470731; doi:10.1371/journal.pone.0179469)
Supplement: S1 Methods — (DOCX) [file pone.0179469.s002.docx]

**S1 Methods**

**Bisulfite direct sequencing**

We used the bisulfite direct sequencing PCR (BSP) method to detect the methylation status of two fragments of Ntf3 promoter region. DNA from mPFC was extracted using DNeasy Tissue System (Qiagen, Inc., Valencia, USA), according to the manufacturer’s instructions. Bisulfite modification of the genomic DNA was carried out using EZ DNA Methylation-Gold Kit (ZYMO RESEARCH). The modified DNA was amplified by PCR. The primers were designed for the Ntf3 promoter using MethPrimer (Ntf3-1-F: TGGGTGTAGAATTGGGGTTTTTA，Ntf3-1-R:ACTCCTCTCCTTACCCAACATCTC; Ntf3-2-F: TGGAGATGTTGGGTAAGGAGAG， Ntf3-2-R: TTCCCTTTAATCTCAAAAACAAACTC ) and 1 µl template DNA in a volume of 20μl. The PCR conditions were 95 ℃ for 2 min, 36 cycles of 94 ℃ for 20 s, 58 ℃ for 40 s, and 72 ℃ for 1 min, with a final extension reaction at 72 ℃ for 2 min. The PCR products were sequenced on an ABI automated sequencer with Dye terminators (Perkin–Elmer Corp., Foster City, CA).

**Quantitation of methylation levels and statistical analysis**

Quantification of methylation levels was performed by comparing the peak height of the cytosine signal with the peak height of the thymine signal. A single “C” at the corresponding CpG site was considered as 100% methylation, a single “T” as no methylation, and overlapping “C” and “T” as partial methylation. In the last case, the percentage of methylation was expressed as the ratio of “C” peak value to the peak values of “C” plus “T.” Data are given as mean±SEM. Statistical analysis was performed using two-way ANOVA. Significance was defined as p *<* 0.05.
